# Ashwagandha Root Extract and Its Potential Modulation of CCN1‐Associated Pathways in Sensitive‐Skin Aging

**DOI:** 10.1111/jocd.71010

**Published:** 2026-06-23

**Authors:** Liying Kang, Yanhong Liu, Yuchen Xin, Lin Geng, Shaochun Cai, Jianfang Luo, Shaojie Gu, Shuangyan Wang, Zhiting Zhang, Yan Huang, Guixuan Zhou, Li Ye, Dongcui Li, Naisheng Jiang

**Affiliations:** ^1^ Key Laboratory of Advanced Materials and Devices for Post‐Moore Chips, Ministry of Education, School of Materials Science and Engineering University of Science and Technology Beijing Beijing China; ^2^ Hua An Tang Biotech Group Co., Ltd. Guangzhou China; ^3^ Skin Lane (Guangzhou) Biotech Co., Ltd. Guangzhou China; ^4^ College of Biological Science and Engineering Fuzhou University Fuzhou China; ^5^ Dermatology Hospital Southern Medical University Guangzhou China; ^6^ NMPA Key Laboratory for Safety Evaluation of Cosmetics Guangdong Provincial Key Laboratory of Tropical Disease Research Guangzhou China

**Keywords:** Ashwagandha root extract, CCN1, oxidative stress, sensitive skin, skin aging

## Abstract

**Background:**

Cellular Communication Network Factor 1 (CCN1) has been identified as a key regulator of oxidative stress‐induced skin aging and sensitivity. Ashwagandha root extract (ARE) is a promising candidate for modulating CCN1‐associated pathways.

**Aims:**

To investigate whether ARE can mitigate sensitive‐skin aging by modulating CCN1 expression and its downstream effects, and to evaluate the clinical efficacy and tolerability of an ARE‐containing formulation in individuals with sensitive skin.

**Methods:**

Human foreskin fibroblasts (HFF‐1) exposed to hydrogen peroxide (H_2_O_2_) were assessed for viability using the Cell Counting Kit‐8 (CCK‐8) assay, and for CCN1, integrin α_6_β_1_, MMP‐1, COL‐1, IL‐6, and IL‐1β expression using enzyme‐linked immunosorbent assay (ELISA). A 28‐day open‐label clinical study was performed in 31 Chinese women with sensitive skin to evaluate changes in skin hydration, elasticity, firmness, wrinkle severity, and facial contour.

**Results:**

H_2_O_2_ exposure significantly decreased fibroblast viability and upregulated CCN1 expression. Treatment with ARE dose‐dependently restored cell viability, suppressed the expression of CCN1, integrin α_6_β_1_, MMP‐1, IL‐6, and IL‐1β, and promoted COL‐1 synthesis. Clinical evaluation demonstrated that 28 days of ARE‐containing cream application significantly improved skin hydration (+49.0%) and elasticity (+27.2%), improved skin firmness, as evidenced by a 32.4% reduction in the F4 parameter compared with baseline (*p* < 0.001), and reduced wrinkle depth and facial sagging.

**Conclusion:**

ARE can alleviate oxidative stress‐induced skin aging at least in part by downregulating CCN1 and its downstream inflammatory mediators and by inhibiting matrix‐metalloproteinase‐mediated extracellular matrix (ECM) degradation. The combined biological and clinical evidence supports ARE as a promising and well‐tolerated active ingredient for improving signs of skin aging in individuals with sensitive skin.

## 1 Introduction

Skin aging arises from both intrinsic and extrinsic influences, yet these pathways converge on several core biological processes, including oxidative stress, low‐grade inflammation, and extracellular matrix (ECM) degradation [1, 2, 3, 4]. These processes promote fibroblast senescence, reduce collagen synthesis, fragment dermal structure, and impair tissue repair, ultimately contributing to visible aging phenotypes such as wrinkles, laxity, and loss of firmness [5, 6, 7]. Among these mechanisms, oxidative stress is widely recognized as a principal upstream driver. Elevated reactive oxygen species (ROS) initiate inflammatory cascades, activate matrix‐degrading enzymes, accelerate senescence‐associated changes, and weaken barrier function, thereby establishing a central axis that links environmental exposures to structural and functional decline in aged skin [4].

Sensitive skin is a common dermatological condition characterized by unpleasant sensations, such as stinging, burning, itching, or tightness, that occur in response to stimuli that ordinarily should not provoke discomfort [8, 9]. Individuals with sensitive skin often exhibit exaggerated reactions to cosmetics, weather changes, and environmental irritants, particularly under dry or cold conditions [10, 11]. Increasing evidence indicates that disrupted barrier organization, combined with heightened neurosensory and vascular reactivity, contributes to this exaggerated cutaneous responsiveness [12, 13, 14]. These alterations may also amplify oxidative stress and inflammatory signaling, thereby linking sensitive skin with biological pathways implicated in premature aging [15, 16, 17]. As a result, sensitive skin may be viewed as a stress‐responsive phenotype with heightened vulnerability to oxidative damage and structural decline [18]. Identifying molecular pathways that integrate oxidative stress, inflammation, and ECM remodeling may thus provide valuable insight for developing targeted anti‐aging strategies suitable for sensitive‐skin populations.

Cellular Communication Network Factor 1 (CCN1, also known as CYR61) is an ECM‐associated matricellular protein that plays a critical regulatory role in dermal homeostasis [19, 20, 21]. Dysregulated CCN1 contributes to impaired barrier integrity, fibroblast senescence, pro‐inflammatory cytokine release, reduced collagen production, and enhanced matrix metalloproteinase activity, collectively driving dermal degradation and microenvironmental imbalance [22, 23, 24, 25]. CCN1 also functions as a mediator of oxidative‐stress responses by activating pathways such as NF‐κB, MAPK, and PI3K/Akt, which further amplify inflammatory and ECM‐remodeling signals [26, 27, 28]. These observations have led to increasing interest in CCN1 as a molecular link between oxidative stress, inflammation, skin sensitivity, and aging. While pharmacological agents such as retinoids can downregulate CCN1 expression and partially restore collagen homeostasis [29], their use is frequently limited in individuals with sensitive skin due to irritation and barrier disruption. This emphasizes the need for alternative strategies capable of modulating CCN1‐associated pathways with improved tolerability.

The implications of CCN1 dysregulation may be further magnified in sensitive skin. Compared with normal skin, sensitive skin is often associated with impaired barrier integrity and heightened neurovascular reactivity, rendering it more susceptible to environmental insults and oxidative stress [9, 10, 30]. This reduced defensive capacity may facilitate the accumulation of reactive oxygen species (ROS) under external challenge [31]. In this context, ROS can act as a key trigger for stress‐responsive mediators such as CCN1, which is involved in inflammatory signaling and extracellular matrix remodeling [25, 32]. Consequently, the stress‐prone microenvironment in sensitive skin may lower the threshold for CCN1 activation, thereby amplifying downstream pro‐inflammatory and matrix‐degrading processes and contributing to accelerated cutaneous aging.

Ashwagandha ( *Withania somnifera* ), an adaptogenic herb widely used in traditional Indian medicine, has long been recognized for its diverse biological activities [33]. Its root contains bioactive withanolides and alkaloids, particularly withaferin A and withanolide A, which exhibit antioxidant, anti‐inflammatory, and anti‐hyperglycemic effects [34, 35, 36]. These activities have generated interest in the potential of Ashwagandha root extract (ARE) to enhance cellular resilience and support extracellular matrix (ECM) homeostasis [34, 37, 38]. A previous study demonstrated that ARE can mitigate methylglyoxal‐induced damage in dermal fibroblasts by modulating ECM‐integrin signaling [38]. However, it remains unknown whether ARE can modulate oxidative‐stress‐induced CCN1 dysregulation or provide clinically meaningful anti‐aging benefits in individuals with sensitive skin.

In this study, we first characterized the chemical composition of ARE using liquid chromatography–tandem mass spectrometry (LC–MS/MS). We then established an H_2_O_2_‐induced oxidative‐stress model in human foreskin fibroblasts (HFF‐1) to examine whether ARE modulates CCN1 expression and downstream inflammatory and ECM‐related pathways. Finally, we evaluated the clinical efficacy and tolerability of an ARE‐containing cream in individuals with sensitive skin through a 28‐day open‐label study. These integrated approaches aimed to investigate the potential of ARE as a CCN1‐associated adaptogenic ingredient for mitigating oxidative‐stress‐related skin aging and improving skin condition in sensitive‐skin populations.

## 2 Materials and Methods

### 2.1 Sample Preparation and Characterization of Ashwagandha Root Extract (ARE)

Dried and cleaned roots of *Withania somnifera* (Ashwagandha) were pulverized into a fine powder. The powder was extracted with hot water at a ratio of 1:25 (w/v) at 80°C for 120 min. The extract was centrifuged and filtered, and the resulting supernatant was concentrated using a nanofiltration membrane to obtain Ashwagandha root extract (ARE). The chemical composition of ARE was characterized by LC–MS/MS (see Supporting Information, Figure S1 and Table S1 for details).

**FIGURE 1:**
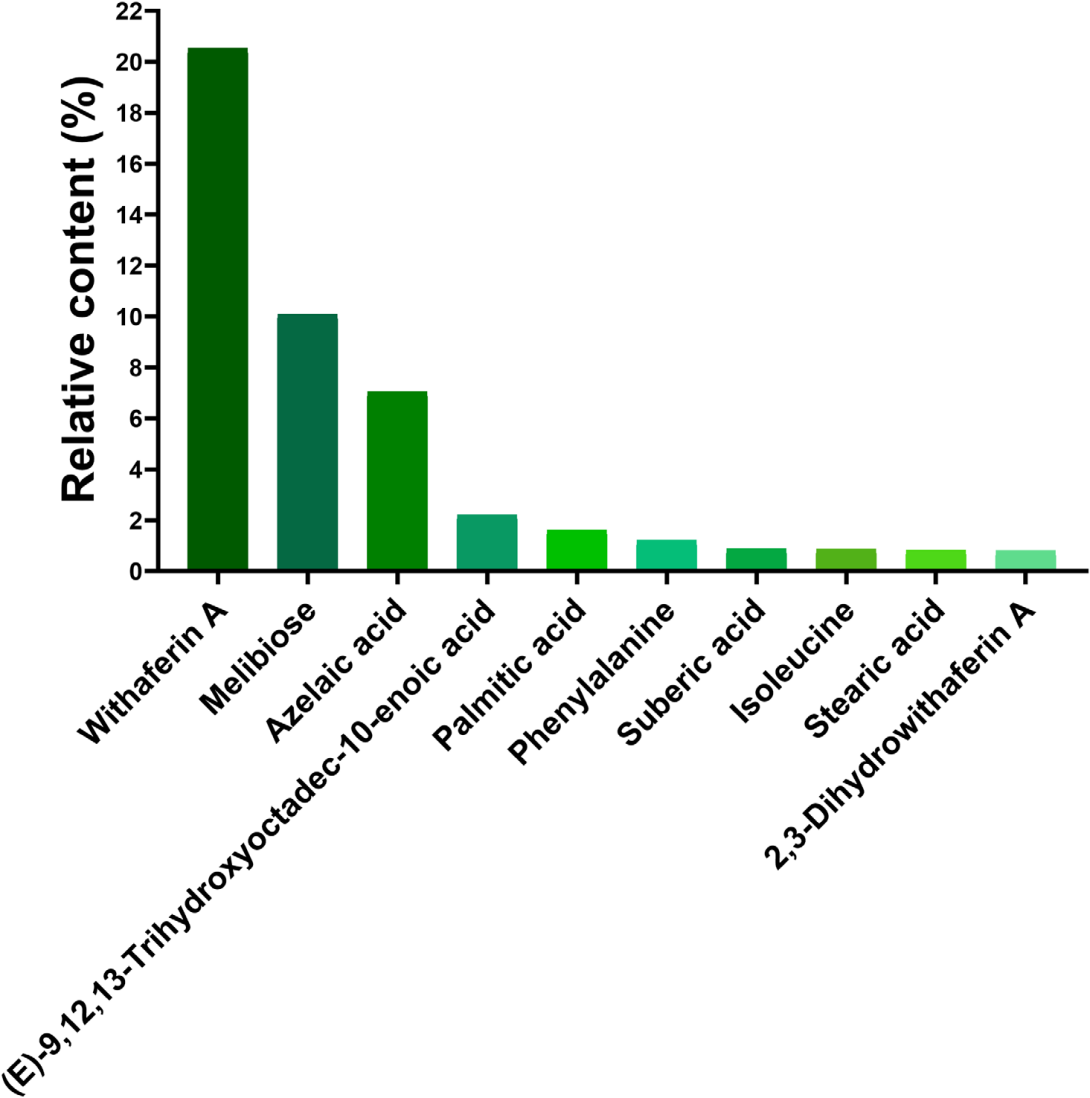
Relative contents of the 10 most abundant compounds identified in ARE.

A total of 45 compounds were identified (Table S1, Supporting Information), including withanolides, alkaloids, terpenoids, organic acids, and other minor phytochemicals. Figure 1 shows the 10 most abundant constituents, among which withanolides predominated, particularly withaferin A and 2,3‐dihydrowithaferin A. It should be noted that withaferin A is designated as a marker compound for *Withania somnifera* in the 2022 Indian Pharmacopeia.

### 2.2 In Vitro Oxidative‐Stress Model

#### 2.2.1 Cell Culture

Human foreskin fibroblasts (HFF‐1; Meisen, China) were cultured in Dulbecco's Modified Eagle Medium (DMEM; CTCC‐002‐008, Meisen, China) supplemented with 15% fetal bovine serum (FBS; CTCC‐002‐071, Meisen, China), 200 kU/L penicillin sodium, and 100 mg/L streptomycin (XA4122, BIOAGRIO, China). Cells were maintained at 37°C in a humidified incubator with 5% CO_2_. All experiments were performed using cells in the logarithmic growth phase.

#### 2.2.2 Establishment and Optimization of the H_2_O_2_ ‐Induced Oxidative‐Stress Model

HFF‐1 cells were seeded into 96‐well plates at a density of 1 × 10^4^ cells/well in complete DMEM and incubated for 24 h at 37°C in a humidified atmosphere containing 5% CO_2_. Oxidative stress was induced by treatment with hydrogen peroxide (H_2_O_2_; H112519‐500, Aladdin, China). To determine the optimal concentration for model establishment, cells were exposed to H_2_O_2_ at concentrations ranging from 0.20 to 0.24 mM for 24 h.

Cell viability was assessed using the Cell Counting Kit‐8 (CCK‐8; BN15201, Biorigin, China). Briefly, 110 μL of CCK‐8 working solution (CCK‐8 reagent diluted 1:10 in serum‐free DMEM) was added to each well and incubated for 1 h at 37°C in the dark. Absorbance was then measured at 450 nm using a microplate reader (Infinite 200 PRO, TECAN, Switzerland).

Cellular senescence was evaluated using senescence‐associated β‐galactosidase (SA‐β‐gal) staining (C0602, Beyotime, China) according to the instructions by the manufacturer. Images were captured at 10× magnification using an inverted fluorescence microscope (IX73, Olympus, Japan). CCN1 protein levels were quantified by ELISA and normalized to total protein content determined by BCA assay (see Supporting Information for details). The optimal H_2_O_2_ concentration was selected based on a combination of reduced cell viability, increased SA‐β‐gal staining, and elevated normalized CCN1 expression.

#### 2.2.3 Determination of Non‐Cytotoxic Treatment Concentrations of ARE

To determine non‐cytotoxic concentrations of Ashwagandha root extract (ARE), HFF‐1 cells were seeded into 96‐well plates at a density of 1 × 10^4^ cells/well and incubated for 24 h. ARE was serially diluted in complete culture medium to final concentrations ranging from 0.05% to 2.0%, and cells were treated for 24 h. After treatment, the medium was removed, and 110 μL of CCK‐8 working solution (1:10 dilution in serum‐free DMEM) was added to each well. Following incubation for 1 h at 37°C in the dark, absorbance was measured at 450 nm using a microplate reader. Cell viability was calculated relative to untreated controls. ARE concentrations maintaining ≥ 80% viability were considered non‐cytotoxic and selected for subsequent experiments.

#### 2.2.4 Effect of ARE on Cell Viability in the H_2_O_2_ Model

HFF‐1 cells were seeded into 96‐well plates at a density of 1 × 10^4^ cells/well and incubated for 24 h. Cells were divided into three groups, that is, a blank control (culture medium only), a model control (H_2_O_2_ treatment only), and treatment groups receiving ARE. Cells in the treatment groups were preincubated with ARE at three non‐cytotoxic concentrations for 1 h without medium replacement. Subsequently, H_2_O_2_ was added at the optimized concentration determined in Section 2.2.2 to induce oxidative stress. Cells were then incubated for an additional 24 h under standard culture conditions. Following treatment, the medium was removed, and 110 μL of CCK‐8 working solution (1:10 dilution in serum‐free DMEM) was added to each well. After incubation for 1 h at 37°C in the dark, absorbance was measured at 450 nm. Cell viability was calculated based on the obtained optical density (OD) values.

### 2.3 Clinical Evaluation of Efficacy and Skin Tolerability of ARE

#### 2.3.1 Topical Cream Containing ARE

A topical oil‐in‐water cream containing Ashwagandha root extract (ARE) as the active ingredient was prepared for clinical evaluation. The formulation comprised standard cosmetic excipients, including emollients, humectants, fatty alcohols, and stabilizing agents, and did not include additional anti‐aging actives/ingredients, allowing clear attribution of observed effects to ARE. The product was manufactured under cosmetic Good Manufacturing Practice (GMP) conditions. Detailed information on the ingredient composition of the product, together with the key excipients of the base O/W cream vehicle, is provided in the Supporting Information (Sections S3 and S4, Table S2).

#### 2.3.2 Clinical Study Design and Assessments

A single‐center, 28‐day, open‐label study was conducted in healthy Chinese women with clinically sensitive skin and visible signs of facial aging. A total of 31 participants aged 20–55 years were recruited in China. All participants met standardized criteria for sensitive skin (Table S3) and exhibited positive responses in a validated 5% lactic‐acid stinging test (Table S4). Eligible subjects were additionally required to present with at least grade‐2 wrinkles (nasolabial folds, crow's feet, or under‐eye wrinkles) according to a validated photoaging atlas, together with visible skin dryness or laxity. All participants underwent a safety patch test and provided written informed consent prior to enrollment. The study was conducted in accordance with the Declaration of Helsinki and the principles of Good Clinical Practice.

Participants applied the ARE‐containing cream twice daily for 28 consecutive days. Clinical assessments were performed at baseline (D_0_), Day 7 (D_7_), and Day 28 (D_28_), and included standardized facial imaging, noninvasive instrumental measurements, and tolerability evaluations. Additional self‐assessment questionnaires were completed on Day 14 (D_14_) and Day 21 (D_21_). A detailed description of all measurements and assessment methods is provided in Table S5 and the Supporting Information.

## 3 Results

### 3.1 ARE Protects HFF‐1 Fibroblasts From Oxidative Stress‐Induced Cytotoxicity

**FIGURE 2:**
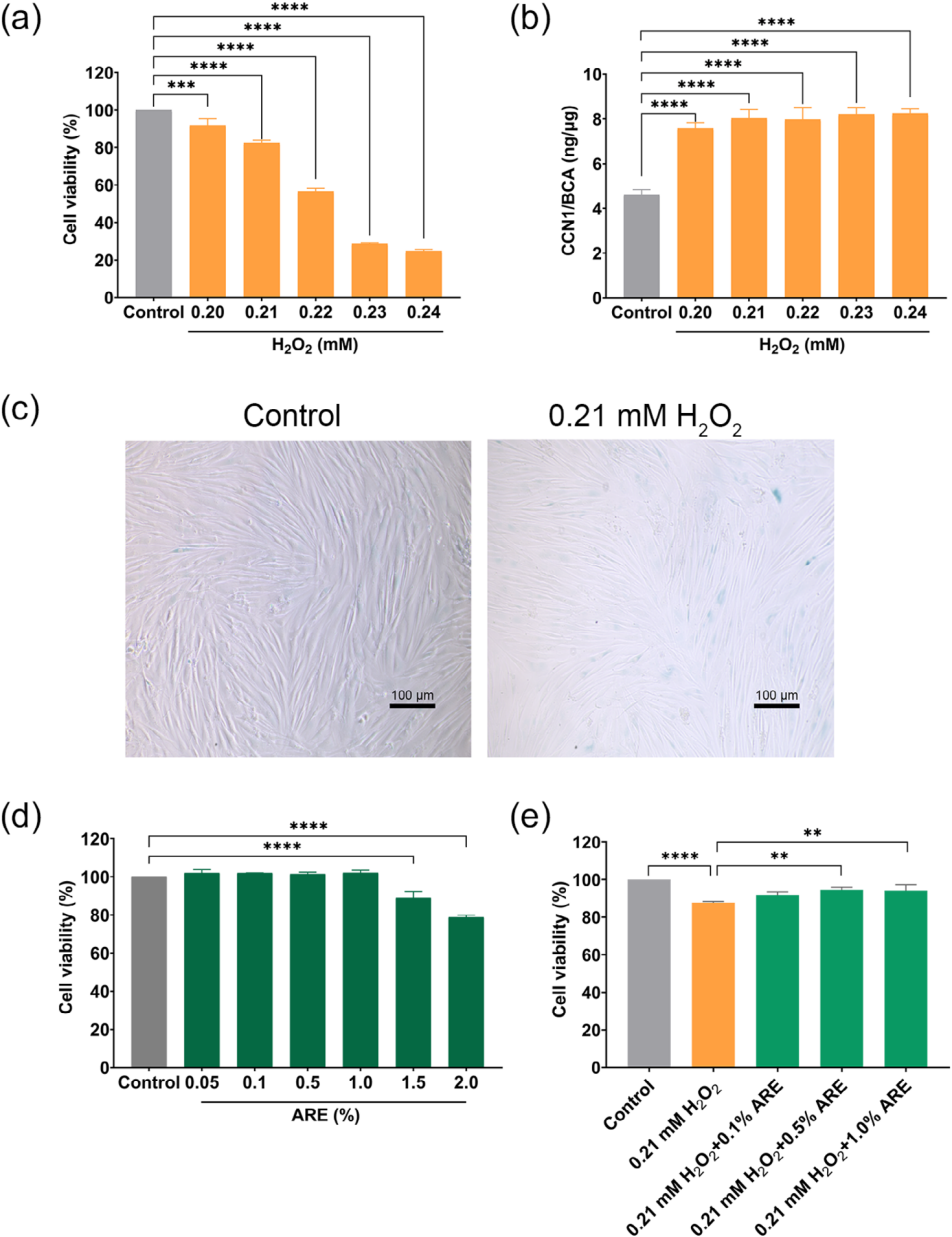
H_2_O_2_‐induced cytotoxicity and the protective effects of ARE in HFF‐1 fibroblasts. (a) Cell viability after treatment with different concentrations of H_2_O_2_. (b) CCN1 protein levels in fibroblasts treated with different concentrations of H_2_O_2_. (c) Representative SA‐β‐gal staining images of untreated fibroblasts and cells exposed to 0.21 mM H_2_O_2_. (d) Cell viability of fibroblasts treated with different concentrations of ARE. (e) Cell viability of H_2_O_2_‐treated fibroblasts following ARE treatment (0.1%–1.0%). Data are presented as mean ± SD (*n* = 3). *p < 0.05, **p < 0.01, ****p < 0.0001 versus control or H_2_O_2_‐treated group.

Human foreskin fibroblasts (HFF‐1) were exposed to increasing concentrations of H_2_O_2_ (0.20–0.24 mM) for 24 h to establish an oxidative stress model. A concentration‐dependent decrease in cell viability was observed (Figure 2a), and 0.21 mM H_2_O_2_ reduced viability to approximately 82.5% of control levels. This concentration was selected for subsequent experiments as it induced measurable oxidative stress without excessive cytotoxicity. At 0.21 mM, CCN1 protein expression increased from 4.6 ng/μg to 8.0 ng/μg total protein, as quantified by ELISA and normalized by BCA assay (Figure 2b). In parallel, senescence‐associated β‐galactosidase (SA‐β‐gal) staining revealed a significant increase in senescent cells relative to untreated controls (Figure 2c). These findings confirmed that exposure to 0.21 mM H_2_O_2_ for 24 h effectively established an oxidative stress‐induced aging model characterized by elevated CCN1 expression and cellular senescence.

To determine the non‐cytotoxic treatment range of ARE, fibroblasts were incubated with 0.05%–2.0% ARE for 24 h. Cell viability remained above 80% at concentrations ≤ 1.0%, whereas 1.5% ARE significantly reduced viability (*p* < 0.0001 vs. control, Figure 2d). Based on these results, 0.1%, 0.5%, and 1.0% ARE were selected for further analysis. In the established oxidative stress model (0.21 mM H_2_O_2_), treatment with 0.5% and 1.0% ARE significantly increased fibroblast viability compared with the H_2_O_2_ group (*p* < 0.01, Figure 2e), whereas 0.1% ARE did not produce a significant effect (*p* > 0.05). These results indicate that ARE provides dose‐dependent protection against H_2_O_2_‐induced cytotoxicity.

### 3.2 ARE Restores Fibroblast and ECM Homeostasis Under Oxidative Stress

**FIGURE 3:**
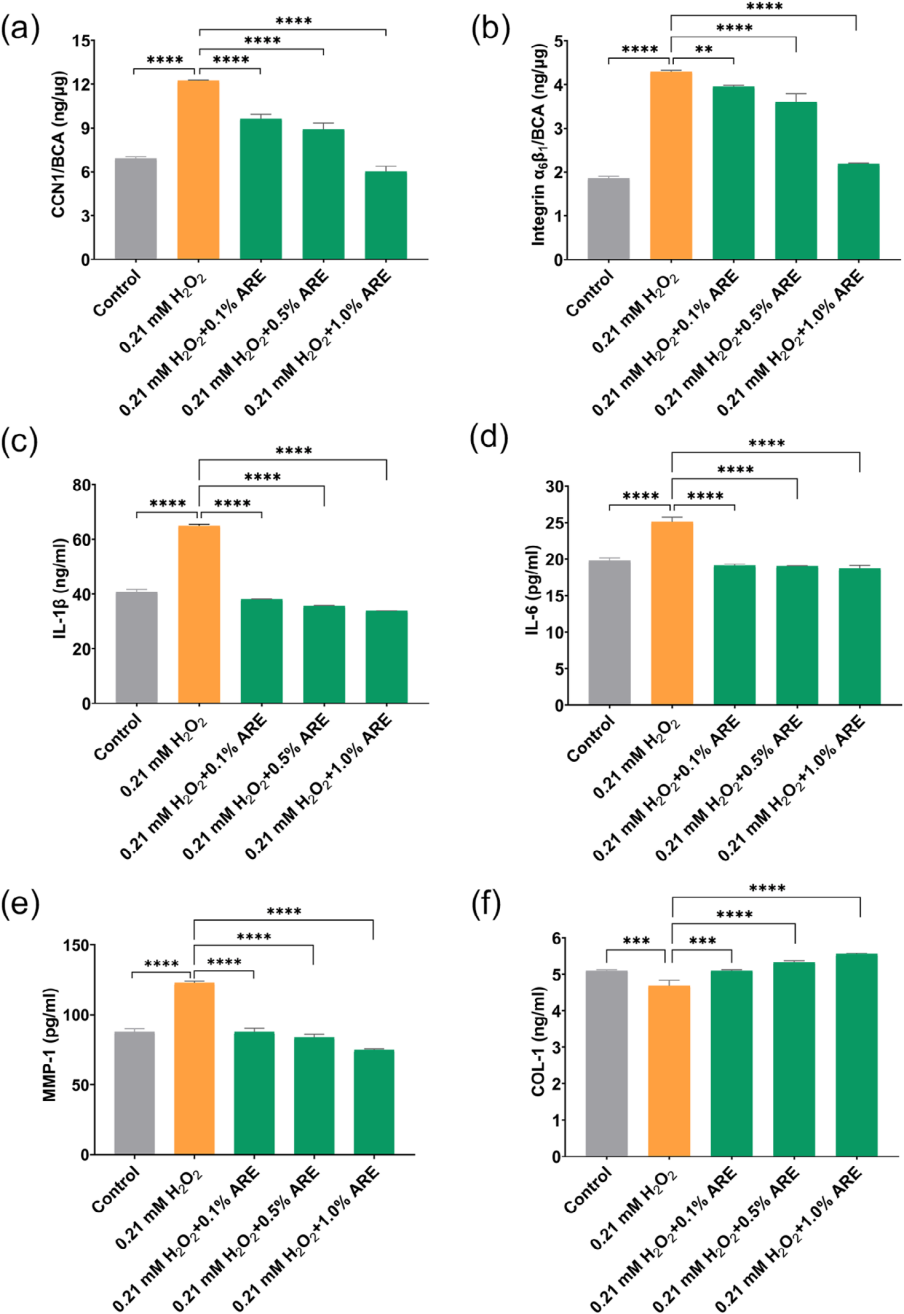
Effects of ARE on senescence‐ and ECM‐related protein expression in H_2_O_2_‐ treated HFF‐1 fibroblasts. (a) CCN1 expression, (b) integrin α_6_β_1_, (c) IL‐1β, (d) IL‐6, (e) MMP‐1, (f) COL‐1. Data are presented as mean ± SD (*n* = 3). *p < 0.05, **p < 0.01, ***p < 0.001, ****p < 0.0001 versus H_2_O_2_ model group.

Beyond reducing cytotoxicity, H_2_O_2_ exposure increased several senescence‐ and inflammation‐related markers in HFF‐1 fibroblasts compared with untreated controls (Figure 3). Specifically, CCN1 expression increased from approximately 6.9 ng/μg in the control group to 12.3 ng/μg following H_2_O_2_ treatment (Figure 3a). ARE reduced CCN1 in a dose‐dependent manner, decreasing levels by 21.3%, 27.2%, and 50.9% at 0.1%, 0.5%, and 1.0% ARE, respectively (*p* < 0.0001). A similar trend was observed for integrin α_6_β_1_, which was elevated after H_2_O_2_ exposure and subsequently reduced by 7.9%, 16.2%, and 49.0% in response to 0.1%, 0.5%, and 1.0% ARE, respectively (*p* < 0.01 or *p* < 0.0001) (Figure 3b). H_2_O_2_ also increased the secretion of pro‐inflammatory cytokines IL‐1β and IL‐6. IL‐1β increased to 65.0 pg/mL and IL‐6 to 25.1 pg/mL compared with 40.8 pg/mL and 19.8 pg/mL in the control group (Figure 3c,d). ARE attenuated this inflammatory response in a dose‐dependent manner, with the 1.0% ARE group showing the largest reductions (48.0% for IL‐1β and 25.4% for IL‐6, *p* < 0.0001). Markers of ECM remodeling were also significantly altered under oxidative stress. H_2_O_2_ increased MMP‐1 to 122.8 pg/mL and decreased COL‐1 to 4.7 ng/mL (Figure 3e,f), indicating ECM degradation and impaired collagen synthesis. ARE reversed these changes in a dose‐dependent manner. Relative to the model group, MMP‐1 levels decreased by 29.2%, 32.6%, and 39.7%, while COL‐1 increased by 9.0%, 13.8%, and 18.6% following treatment with 0.1%, 0.5%, and 1.0% ARE, respectively (*p* < 0.001 or *p* < 0.0001). These data demonstrate that ARE can mitigate oxidative‐stress‐induced senescence and inflammation, while restoring ECM‐related protein expression in HFF‐1 fibroblasts.

### 3.3 Clinical Results of ARE‐Containing Cream

Sensitive skin is characterized by impaired barrier function, reduced hydration, and heightened sensory reactivity [9, 18], making it a suitable model for evaluating topical agents aimed at improving skin resilience. Based on the in vitro findings that ARE supported fibroblast and ECM homeostasis under oxidative stress, a 28‐day clinical study was conducted to assess the efficacy and tolerability of a topical cream containing 1.0% ARE in individuals with sensitive skin. Preliminary screening also showed that 1.0% ARE reduced capsaicin‐induced stinging sensations (Figure S2).

#### 3.3.1 Clinical Safety Assessment in Sensitive Skin Subjects

Across the 28‐day trial involving 31 participants with clinically confirmed sensitive skin (lactic‐acid stinging score ≥ 3), the 1.0% ARE cream demonstrated good overall tolerability. No adverse events or signs of irritation were reported during the study. Participant self‐assessments also indicated good comfort and compatibility throughout the treatment period.

#### 3.3.2 Improvements in Skin Biophysical Properties

**FIGURE 4:**
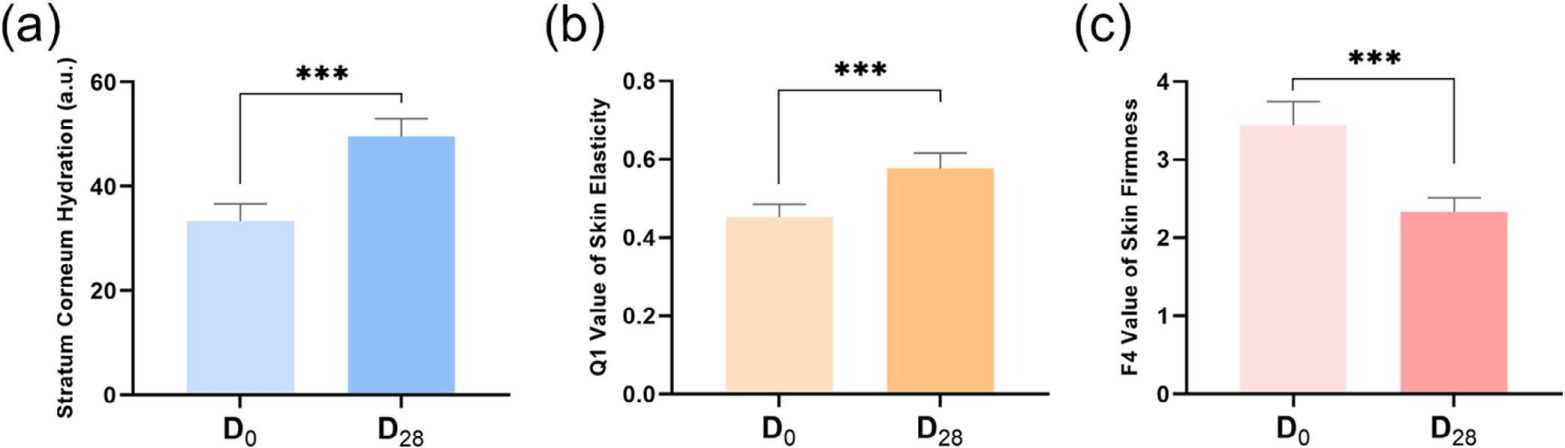
Biophysical skin parameters measured at baseline (D_0_) and after 28 days (D_28_) of twice‐daily application of the ARE‐containing cream. (a) Stratum corneum hydration; (b) skin elasticity (Q1 value); (c) skin firmness (F4 value). All parameters showed significant improvement compared with baseline (***p < 0.001).

As shown in Figure 4, twice‐daily application of the ARE‐containing cream for 28 days led to significant improvements in key biophysical parameters associated with barrier function and skin biomechanics. It was found that stratum corneum hydration increased by 49.0% (Figure 4a), while skin elasticity (Q1 value) improved by 27.2% (Figure 4b). The firmness parameter (F4 value) decreased by 32.4% (Figure 4c), suggesting improved skin firmness and elasticity. All changes were statistically significant (*p* < 0.001), demonstrating that ARE contributed to measurable enhancement of hydration, elasticity, and overall biomechanical performance in individuals with sensitive skin.

#### 3.3.3 Clinical Anti‐Wrinkle Efficacy in Multiple Facial Regions

As summarized in Table 1, instrumental assessments showed that 28 days of application of the ARE‐containing cream resulted in significant reductions in multiple wrinkle parameters across the nasolabial folds (NLFs), infraorbital wrinkles (IOWs), and crow's‐feet wrinkles (CFWs). Wrinkle number decreased by 33.3% for both NLFs and IOWs and by 25.9% for CFWs, with improvement observed in 93.5%–100.0% of participants. Wrinkle length was also reduced by 16.9% (NLFs), 17.0% (IOWs), and 25.5% (CFWs), and mean wrinkle width decreased by 11.6% in NLFs.

**TABLE 1:** Instrumental analysis of facial wrinkle parameters.

Item	Parameter	NLFs	IOWs	CFWs
Wrinkle number	Reduction rate (%)	33.3	33.3	25.9
	Subjects with improvement (%)	96.8	100.0	100.0
	*p* value	< 0.001	< 0.001	< 0.001
Wrinkle length	Reduction rate (%)	16.9	17.0	25.5
	Subjects with improvement (%)	96.8	100.0	100.0
	*p* value	< 0.001	< 0.001	< 0.001
Mean width	Reduction rate (%)	11.6	N/A	N/A
	Subjects with improvement (%)	100.0	N/A	N/A
	*p* value	< 0.001	N/A	N/A
Wrinkle area	Reduction rate (%)	N/A	26.2	27.2
	Subjects with improvement (%)	N/A	100.0	100.0
	*p* value	N/A	< 0.001	< 0.001
Mean depth	Reduction rate (%)	14.9	14.9	21.0
	Subjects with improvement (%)	96.8	100.0	100.0
	*p* value	< 0.001	< 0.001	< 0.001
Surface roughness	Reduction rate (%)	29.2	29.2	15.2
	Subjects with improvement (%)	100.0	96.8	100.0
	*p* value	< 0.001	< 0.001	< 0.001

*Note:* (1) Reduction rate (%) = (baseline–posttreatment)/baseline × 100%. (2) Subjects with improvement (%) = (number of subjects showing improvement/total subjects) × 100%.

**FIGURE 5:**
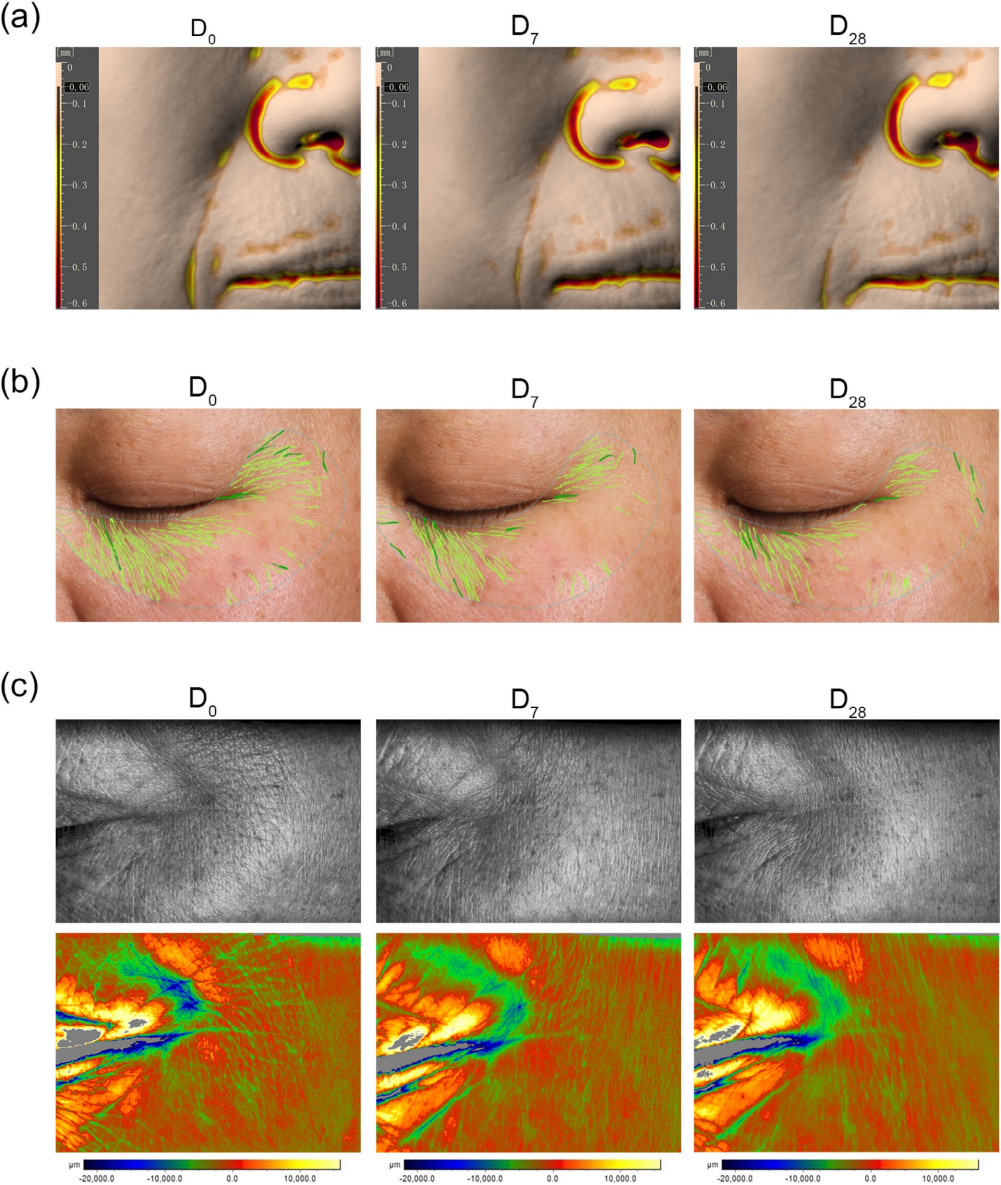
Representative clinical photographs showing wrinkle improvement after 28 days of application of the ARE‐containing cream across three facial regions: (a) nasolabial folds (NLFs), (b) infraorbital wrinkles (IOWs), and (c) crow's‐feet wrinkles (CFWs). Images were captured at Day 0 (D_0_, baseline), Day 7 (D_7_), and Day 28 (D_28_).

It was also found that wrinkle area declined by 26.2% (IOWs) and 27.2% (CFWs), indicating reduced wrinkle surface coverage. Mean wrinkle depth decreased by 14.9% in NLFs and IOWs and by 21.0% in CFWs. Surface roughness, an indicator of overall skin smoothness, was also reduced by 29.2% (NLFs), 29.2% (IOWs), and 15.2% (CFWs). Across all parameters, improvements were statistically significant (*p* < 0.001) and were observed in at least 96.8% of participants. Representative clinical photographs were consistent with these instrumental findings, showing progressive softening of wrinkles from Day 0 to Day 7 and more pronounced improvements by Day 28 (Figure 5). In all three facial regions, wrinkles appeared shallower with smoother contours and improved surface uniformity, consistent with the quantitative results.

#### 3.3.4 Effects on Facial Contour

**FIGURE 6:**
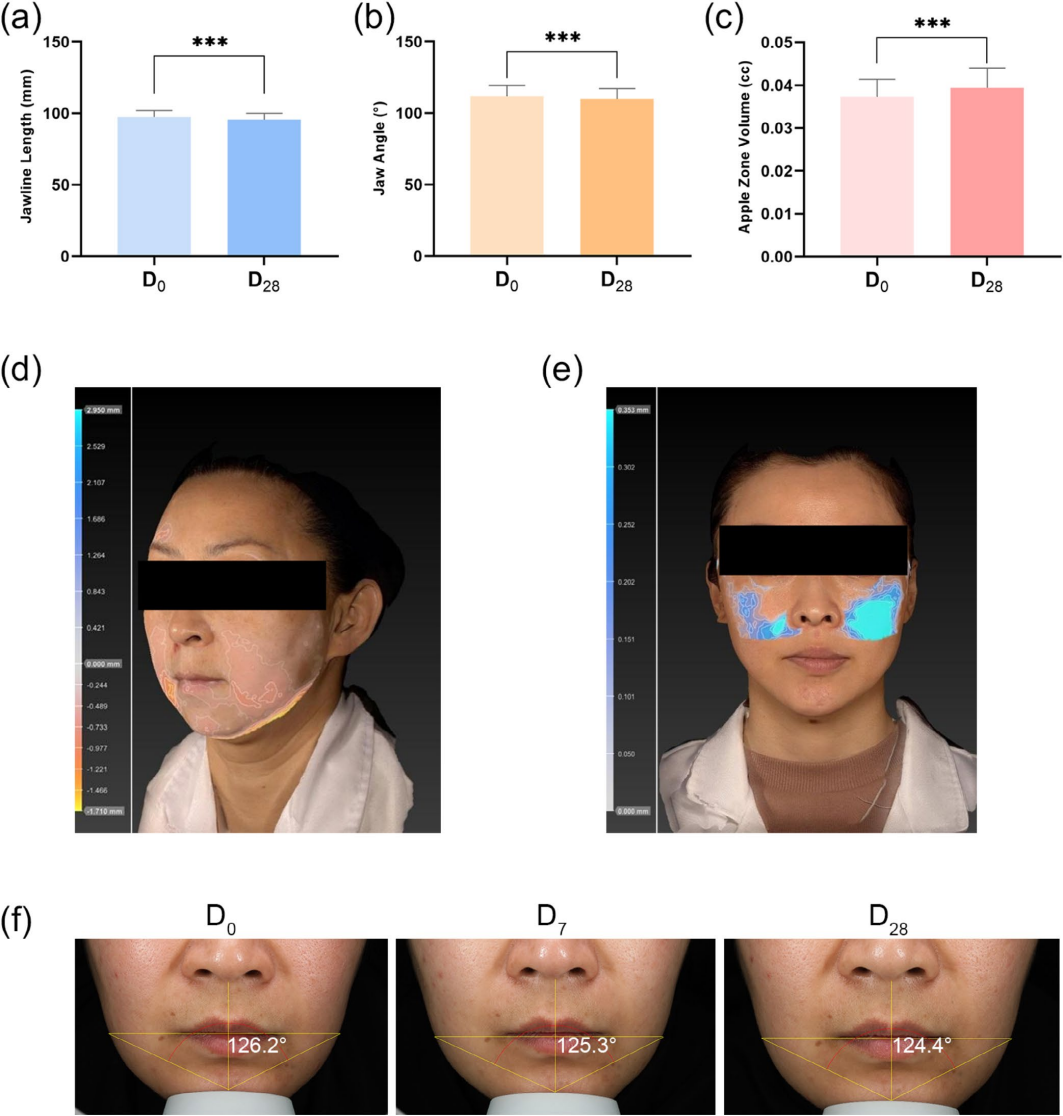
Clinical effects of the ARE‐containing cream on facial contour after 28 days of application. (a) Jawline length, (b) jaw angle, and (c) apple zone volume measured at baseline (D_0_) and Day 28 (D_28_). (d) VECTRA H2 jawline image, (e) VECTRA H2 apple zone image, and (f) VISIA jaw angle image.

After 28 days of twice‐daily application of the ARE‐containing cream, quantitative facial contour measurements showed statistically significant improvements. It was found that jawline length decreased by 2.0% (Figure 6a), jaw angle decreased by 1.6% (Figure 6b), and apple zone volume was increased by 5.7% (Figure 6c), which were all statistically significant (*p* < 0.001). These dimensional changes indicate enhanced contour definition, consistent with a visually lifted and more youthful appearance. Representative images captured using the VECTRA H2 and VISIA 7 systems further supported these results. As shown in Figure 6d–f, treated subjects exhibited a visibly shorter jawline, a reduced mandibular angle, and improved apple zone profile at Day 28 compared with baseline (D_0_). These observations are in line with the instrumental measurements, demonstrating that the ARE‐containing cream contributes to measurable and visible refinements in facial contour and overall facial harmony.

## 4 Discussion

In this study, H_2_O_2_‐induced oxidative stress significantly increased CCN1 expression in human foreskin fibroblasts (HFF‐1), consistent with its established role as a matricellular regulator of oxidative damage and cellular senescence. Oxidative stress is a central driver of skin aging and can result from UV radiation, pollution, cigarette smoke, mitochondrial dysfunction, and chronic inflammation [1, 39, 40]. These stimuli generate excessive reactive oxygen species (ROS), including superoxide (O_2_^−^), hydrogen peroxide (H_2_O_2_), and hydroxyl radicals (•OH), which can disrupt redox balance and damage cellular macromolecules [41]. Numerous studies report that oxidative stress strongly upregulates CCN1 [25, 42]. Once induced, CCN1 interacts with integrins (e.g., α_v_β_3_, α_6_β_1_) and heparan sulfate proteoglycans (HSPGs), activating downstream signaling pathways including NF‐κB, p38 MAPK, and PI3K/Akt [43, 44, 45, 46]. These pathways can increase pro‐inflammatory cytokines and matrix‐degrading enzymes, accelerating type I and III collagen degradation and compromising dermal structural integrity [23]. Furthermore, CCN1 can also suppress collagen synthesis and induce fibroblast cell‐cycle arrest, thereby amplifying senescence under chronic oxidative stress [47, 48]. In addition, persistent oxidative and inflammatory signaling may sensitize cutaneous nerves and microvasculature, leading to neurovascular hyperreactivity and contributing to premature aging in sensitive‐skin phenotypes [17, 49]. In fact, our findings align with these mechanisms. In HFF‐1 fibroblasts, H_2_O_2_ exposure significantly increased CCN1, integrin α_6_β_1_, IL‐1β, IL‐6, and MMP‐1, while decreasing COL‐1. These data further support the central role of CCN1 in regulating oxidative‐stress responses and promoting ECM degradation.

**FIGURE 7:**
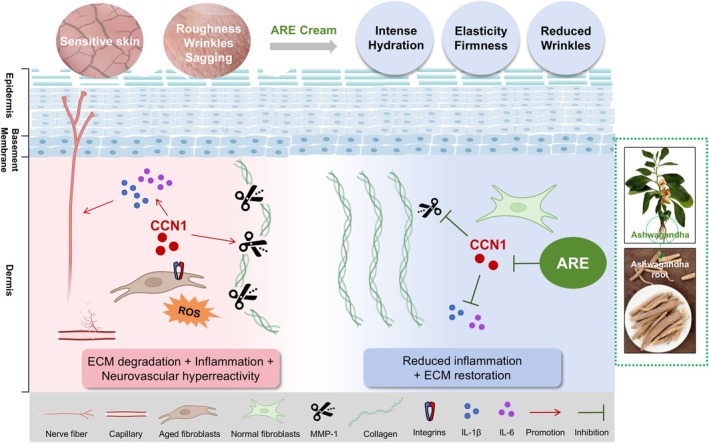
Schematic illustration of the proposed cellular and skin‐level pathways through which ARE may protect against oxidative‐stress‐induced skin aging. Oxidative stress upregulates CCN1, increases inflammatory cytokines and MMP‐1, and reduces COL‐1, contributing to ECM degradation and sensitive‐skin symptoms. On the other hand, ARE downregulates CCN1, decreases inflammatory mediators, inhibits MMP‐1, and restores COL‐1 production, effects that may underlie the observed improvements in skin hydration, elasticity, firmness, and wrinkle appearance.

More importantly, it was found that treatment with ARE effectively reversed these oxidative‐stress‐induced alterations. We postulate that ARE suppressed CCN1 expression and, in turn, reduced inflammatory cytokines (IL‐1β, IL‐6) and MMP‐1, while restoring COL‐1 production. This suggests that ARE preserves ECM homeostasis at least in part through the downregulation of CCN1, a key regulatory node linking oxidative stress, inflammation, and ECM remodeling (Figure 7). Note that similar mechanisms have been reported for retinoids [29] and pterostilbene [50], which also lower CCN1 and promote collagen synthesis, further validating this pathway as a therapeutic target. The comparable effects observed with ARE, along with its demonstrated good tolerability in sensitive skin, highlight its promise as an alternative for sensitive‐skin populations.

At the clinical level, these molecular effects were reflected in measurable improvements in biophysical parameters and overall skin appearance. As aforementioned, after 28 days of twice‐daily application of a topical cream containing 1.0% ARE, participants showed enhanced hydration and elasticity, improved firmness, along with visible reductions in wrinkles and improved facial contour parameters. Improvements in stinging sensations further suggest reduced neurosensory reactivity, an important benefit for sensitive skin. Because sensitive skin is often associated with impaired barrier function and heightened sensory responsiveness [9], these clinical findings indicate strengthened skin resilience, likely due to CCN1 modulation and the subsequent reduction in inflammation and ECM degradation (Figure 7). Together with the absence of irritation or adverse reactions, the findings support 1.0% ARE cream as a suitable and well‐tolerated option for sensitive, aging‐prone skin. It should be noted that Narra et al. [51] previously reported that topical application of an 8% Ashwagandha lotion improved skin hydration and elasticity in photoaged healthy adults over 60 days. Consistent with these findings, our study demonstrated significant improvements in skin hydration and biomechanical properties following 28 days of application of a 1.0% ARE‐containing cream in individuals with sensitive skin. Importantly, beyond clinical efficacy, our results provide support for the involvement of CCN1‐associated pathways, including oxidative stress, inflammatory signaling, and extracellular matrix remodeling, thereby offering additional insight into the potential role of topical Ashwagandha in sensitive‐skin aging.

Despite these encouraging findings, several limitations should be acknowledged. The clinical study involved a relatively small sample size (*n* = 31), a short assessment period (28 days), and a fixed ARE concentration (1.0%), which may limit statistical power, long‐term evaluation, and dose–response assessment. The open‐label design without blinding may introduce observer bias and limits direct comparison with randomized controlled trials. In addition, although multiple biophysical and imaging measurements were performed, certain aspects of facial‐contour improvement relied partly on photographic evaluation. The study population was restricted to Chinese women with sensitive skin, and the efficacy and safety profile therefore warrant validation in broader and more diverse populations, including different ethnic groups, age ranges, and skin types. Moreover, although our in vitro findings support CCN1‐associated modulation under oxidative stress, direct in vivo confirmation of CCN1 regulation in human skin following topical ARE application was not performed.

To address these limitations, larger‐scale, multicenter, randomized, double‐blind, placebo‐controlled trials with extended follow‐up periods (≥ 12 weeks) are needed to confirm the durability and generalizability of the observed benefits. Future studies incorporating 3D volumetric analysis, biomarker profiling, and dose–response evaluation will further strengthen the understanding of ARE's biological effects and optimize its formulation. In addition, detailed studies employing targeted CCN1 modulation and biopsy‐based analyses will be important to clarify the involvement of CCN1‐associated pathways. Clinical studies with extended durations will also be valuable to assess the sustainability of improvements in hydration, elasticity, and wrinkle parameters. Nevertheless, the present findings suggest that ARE is a promising and well‐tolerated skincare ingredient for addressing skin aging in sensitive‐skin populations, potentially through modulation of CCN1‐associated pathways.

## 5 Conclusion

In this study, we demonstrate that Ashwagandha root extract (ARE) can mitigate oxidative‐stress‐induced damage in human foreskin fibroblasts (HFF‐1) and improve aging‐related skin characteristics in individuals with sensitive skin. In vitro, ARE improved the viability of H_2_O_2_‐exposed fibroblasts and suppressed key stress‐associated markers. Specifically, ARE reduced CCN1 overexpression by up to 50.9%, decreased IL‐1β and IL‐6 secretion by 48.0% and 25.4%, respectively, lowered MMP‐1 levels by 39.7%, restored type I collagen (COL‐1) expression, and attenuated senescence‐associated changes. It was also found that 28 days of topical application of a cream containing 1.0% ARE resulted in significant improvements in stratum corneum hydration (+49.0%) and elasticity (+27.2%), together with a 32.4% reduction in the F4 parameter, indicating improved skin firmness. Reductions were observed across multiple wrinkle parameters, including wrinkle number (−33.3%), wrinkle depth (−14.9% to −21.0%), and surface roughness (−15.2% to −29.2%). Facial‐contour measurements also showed measurable improvements, including a 2.0% reduction in jawline length and a 5.7% increase in apple‐zone volume. Participants further reported reduced stinging sensations, reflecting improved sensory comfort, a key benefit for sensitive‐skin populations. No irritation or adverse reactions were observed throughout the study. These findings suggest that ARE may modulate oxidative‐stress‐related processes, particularly those involving CCN1 and extracellular‐matrix remodeling, while topical delivery translates these biological effects into measurable improvements in skin function and appearance. Future studies employing CCN1 knockdown or overexpression models will be valuable for clarifying the involvement of CCN1‐associated pathways in the observed effects.

## Author Contributions

**Liying Kang:** investigation, writing – original draft, writing – review and editing, visualization. **Yanhong Liu:** methodology, writing – review and editing. **Yuchen Xin:** formal analysis, data curation, writing – original draft, writing – review and editing. **Lin Geng:** formal analysis, data curation, writing – original draft, writing – review and editing. **Jianfang Luo:** formal analysis, data curation, writing – original draft, writing – review and editing. **Shaojie Gu:** formal analysis, data curation, writing – original draft. **Shuangyan Wang:** visualization, writing – review and editing. **Zhiting Zhang:** visualization, writing – review and editing. **Shaochun Cai:** formal analysis, data curation, writing – review and editing. **Yan Huang:** writing – review and editing. **Guixuan Zhou:** supervision, writing – review and editing. **Li Ye:** conceptualization, project administration, supervision, writing – review and editing. **Dongcui Li:** conceptualization, project administration, supervision, writing – review and editing. **Naisheng Jiang:** conceptualization, project administration, supervision, writing – review and editing.

## Ethics Statement

Clinical studies were conducted in accordance with the ethical principles of the Declaration of Helsinki and the guidelines for Good Clinical Practice.

## Conflicts of Interest

The authors declare the following financial interests/personal relationships which may be considered as potential competing interests: Authors Yanhong Liu, Yuchen Xin, Lin Geng, Jianfang Luo, Shaojie Gu, Shuangyan Wang, Zhiting Zhang and Dongcui Li were employed by the company Hua An Tang Biotech Group Co. Ltd. Author Shaochun Cai was employed by the company Skin Lane (Guangzhou) Biotech Co. Ltd. The remaining authors declare no conflicts of interest.

## Supporting information


**Data S1:** LC‐MS/MS analysis of Ashwagandha root extract (ARE).
**Data S2:** Measurement of protein expression by ELISA.
**Data S3:** Composition of ARE‐containing cream.
**Data S4:** Base O/W cream vehicle (key excipients).
**Data S5:** Detailed clinical study procedures.
**Data S6:** Outcome measures.
**Data S7:** Statistical analysis.
**Figure S1:** LC–MS/MS chromatogram of ARE.
**Figure S2:** Capsaicin‐induced stinging scores assessed before and after application of distilled water (control) or a 1.0% ARE formulation. Fifteen Asian adults (18–55 years) participated in the study. The test was conducted on the inner forearm under controlled environmental conditions (2°C1 ± 1°C, 50% ± 10% relative humidity). A 200 μL aliquot of 0.1% capsaicin in ethanol was applied under occlusion for 10 min, followed by removal of the patch. Subjects exhibiting a redness induction rate ≥ 40% were included in the analysis. Each test formulation (distilled water or 1.0% ARE) was applied once (25 μL) to the test site after capsaicin challenge. Stinging intensity was rated on a 0–10 scale at baseline, immediately after capsaicin exposure (*t*_0_), and 30 min after formulation application (*t*_30_).
**Table S1:** Chemical composition of Ashwagandha root extract (ARE) identified by LC–MS/MS.
**Table S2:** Key excipients of the base O/W cream vehicle.
**Table S3:** Subjective sensitivity questionnaire scoring criteria.
**Table S4:** Lactic‐acid stinging test scoring criteria.
**Table S5:** Outcome measures and assessment methods.

## Data Availability

The data that support the findings of this study are available from the corresponding author upon reasonable request.
