# Supplementary material for: Ashwagandha Root Extract and Its Potential Modulation of CCN1‐Associated Pathways in Sensitive‐Skin Aging
Source: J Cosmet Dermatol. 2026 Jun 23;25(7):e71010. doi: 10.1111/jocd.71010 (PMC13288310; doi:10.1111/jocd.71010)
Supplement: Supplementary file 1 — Data S1: LC‐MS/MS analysis of Ashwagandha root extract (ARE). Data S2: Measurement of protein expression by ELISA. Data S3: Composition of ARE‐containing cream. Data S4: Base O/W cream vehicle (key excipients). Data S5: Detailed clinical study procedures. Data S6: Outcome measures. Data S7: Statistical analysis. Figure S1: LC–MS/MS chromatogram of ARE. Figure S2: Capsaicin‐induced stinging scores assessed before and after application of distilled water (control) or a 1.0% ARE formulation. Fifteen Asian adults (18–55 years) participated in the study. The test was conducted on the inner forearm under controlled environmental conditions (2°C1 ± 1°C, 50% ± 10% relative humidity). A 200 μL aliquot of 0.1% capsaicin in ethanol was applied under occlusion for 10 min, followed by removal of the patch. Subjects exhibiting a redness induction rate ≥ 40% were included in the analysis. Each test formulation (distilled water or 1.0% ARE) was applied once (25 μL) to the test site after capsaicin challenge. Stinging intensity was rated on a 0–10 scale at baseline, immediately after capsaicin exposure (t 0), and 30 min after formulation application (t 30). Table S1: Chemical composition of Ashwagandha root extract (ARE) identified by LC–MS/MS. Table S2: Key excipients of the base O/W cream vehicle. Table S3: Subjective sensitivity questionnaire scoring criteria. Table S4: Lactic‐acid stinging test scoring criteria. Table S5: Outcome measures and assessment methods. [file JOCD-25-e71010-s001.docx]

**Supporting Information for**

**Ashwagandha Root Extract and Its Potential Modulation of CCN1‑Associated Pathways in Sensitive-Skin Aging**

**Additional Materials and Characterization Methods**

**S1. LC-MS/MS Analysis of Ashwagandha Root Extract (ARE)**

A 100 μL aliquot of ARE was mixed with 400 μL of extraction solvent (methanol:acetonitrile = 1:1, v/v) containing isotopically labeled internal standards in an Eppendorf tube. The mixture was vortexed for 30 s to ensure thorough mixing, followed by ultrasonication in an ice-water bath for 10 min. Samples were then stored at −40 °C for 1 h to promote protein precipitation. Subsequently, samples were centrifuged at 12 000 rpm for 15 min at 4 °C, and the supernatant was transferred to autosampler vials for analysis.

Liquid chromatography-tandem mass spectrometry (LC-MS/MS) analysis was performed using a Vanquish UHPLC system coupled to an Orbitrap Exploris 120 mass spectrometer (Thermo Fisher Scientific). Chromatographic separation was achieved on a Phenomenex Kinetex C18 column (2.1 × 50 mm, 2.6 μm). The mobile phases consisted of (A) 0.01% aqueous acetic acid and (B) isopropanol/acetonitrile (1:1, v/v). The gradient program was as follows: 0-0.5 min, 1% B; 0.5-4.0 min, 1-99% B; 4.0-4.5 min, 99% B; 4.5-4.55 min, 99-1% B; 4.55-6.0 min, 1% B. The flow rate was 0.3 mL/min, column temperature was maintained at 25 ℃, and the injection volume was 2 μL. Data were acquired in both positive and negative ion modes. Compound identification was performed by matching mass spectra against an internal reference database.

**S2. Measurement of Protein Expression by ELISA**

For CCN1 quantification, cells were lysed using a total protein extraction kit (WB2101, Biotechwell, Shanghai, China). Total protein concentrations were determined using a bicinchoninic acid (BCA) assay (WB0123, Biotechwell, Shanghai, China). CCN1 levels were measured using a commercial ELISA kit (M1591147, Mlbio, China) and normalized to total protein content to obtain CCN1/total protein ratios.

Protein levels of integrin α_6_β_1_, MMP-1, COL-1, and IL-6 were quantified using ELISA kits from Mlbio (Shanghai, China; Cat. Nos. M1063737, M1038199, M1057630, and M1053097, respectively). IL-1β levels were measured using an ELISA kit from Cloud-Clone Corp. (Wuhan, China; Cat. No. L240418776). All ELISA assays were performed strictly according to the instructions provided by the manufacturer.

**S3. Composition of ARE-Containing Cream**

The detailed ingredient list of the topical cream formulation is as follows: Aqua, glycerin, dimethicone, butylene glycol, Ashwagandha root extract, pentaerythrityl tetraethylhexanoate, cetearyl alcohol, dipropylene glycol, *Limnanthes alba* (meadowfoam) seed oil, cetyl ethylhexanoate, triethylhexanoin, glyceryl stearate, PEG-100 stearate, betaine, 1,2-hexanediol, dimethicone/vinyl dimethicone crosspolymer, hydroxyacetophenone, cetearyl glucoside, polyvinyl alcohol, acrylates/C10-30 alkyl acrylate crosspolymer, carbomer, arginine, disodium EDTA, and caprylyl glycol.

**S4. Base O/W Cream Vehicle (Key Excipients)**

The clinical test formulation was developed using a standard oil-in-water (O/W) emulsion system. The base vehicle consisted of water, humectants, emollients, emulsifiers, rheology modifiers, a chelating agent, and a preservative system. Ashwagandha root extract (ARE) was incorporated into this base at 1% (w/w) to obtain the final test formulation. Key excipients of the base O/W vehicle (excluding ARE) are listed in Table S2 according to the International Nomenclature of Cosmetic Ingredients (INCI).

**S5. Detailed Clinical Study Procedures**

Sensitive skin was confirmed using a validated subjective questionnaire (cumulative score ≥ 4; scoring criteria shown in Table S3) and a 5% lactic-acid stinging test. During the 5-min stinging test, sensory responses were recorded at 2.5 and 5 min. A cumulative score ≥ 3 relative to the distilled water control was considered positive (evaluation criteria shown in Table S4).

Exclusion criteria included recent use of anti-inflammatory, immunosuppressive, or antihistamine medications; chronic systemic diseases (e.g., diabetes, asthma, autoimmune disorders); pregnancy or lactation; active dermatologic conditions; and participation in other clinical trials within the preceding two months.

All in-clinic assessments were conducted under controlled environmental conditions (21±1 °C; 50±10% relative humidity). Prior to each assessment, participants cleansed their face using a standardized facial cleanser and underwent a 30-min acclimatization period. Instrument-based evaluations included measurements of skin hydration, elasticity, firmness, wrinkle parameters, and facial contour using standardized non-invasive devices. Standardized facial photographs were captured at each visit under consistent lighting, positioning, and imaging settings. Participants also completed structured questionnaires evaluating product tolerability, sensory experience, and perceived efficacy.

**S6. Outcome Measures**

Primary outcome measures included safety, tolerability, and efficacy. Safety and tolerability were assessed via baseline lactic-acid stinging tests, continuous monitoring of adverse events, and participant self-assessment questionnaires. Efficacy was evaluated using non-invasive biophysical instruments to quantify skin hydration, elasticity, firmness, wrinkle parameters, and facial contour, supplemented by standardized clinical imaging. A summary of outcome measures and assessment methods is provided in Table S5.

**S7. Statistical Analysis**

In vitro data are presented as mean ± standard deviation (SD) from at least three independent experiments. Statistical analyses were performed using GraphPad Prism 9 (GraphPad Software, USA). One-way analysis of variance (ANOVA) followed by Tukey’s multiple-comparisons test was applied for normally distributed data. A *p*-value < 0.05 was considered statistically significant. Significance levels are denoted as follows: **p* < 0.05; ***p* < 0.01; ****p* < 0.001; *****p* < 0.0001.

Clinical data were analyzed using SPSS Statistics (version 19.0; IBM Corp., Armonk, NY, USA). Continuous variables are expressed as mean ± SD. Paired t-tests were applied for normally distributed data, while Wilcoxon signed-rank tests were used for non-normally distributed variables. Questionnaire-based self-assessment results were analyzed using binomial tests. A two-tailed *p* < 0.05 was considered statistically significant.


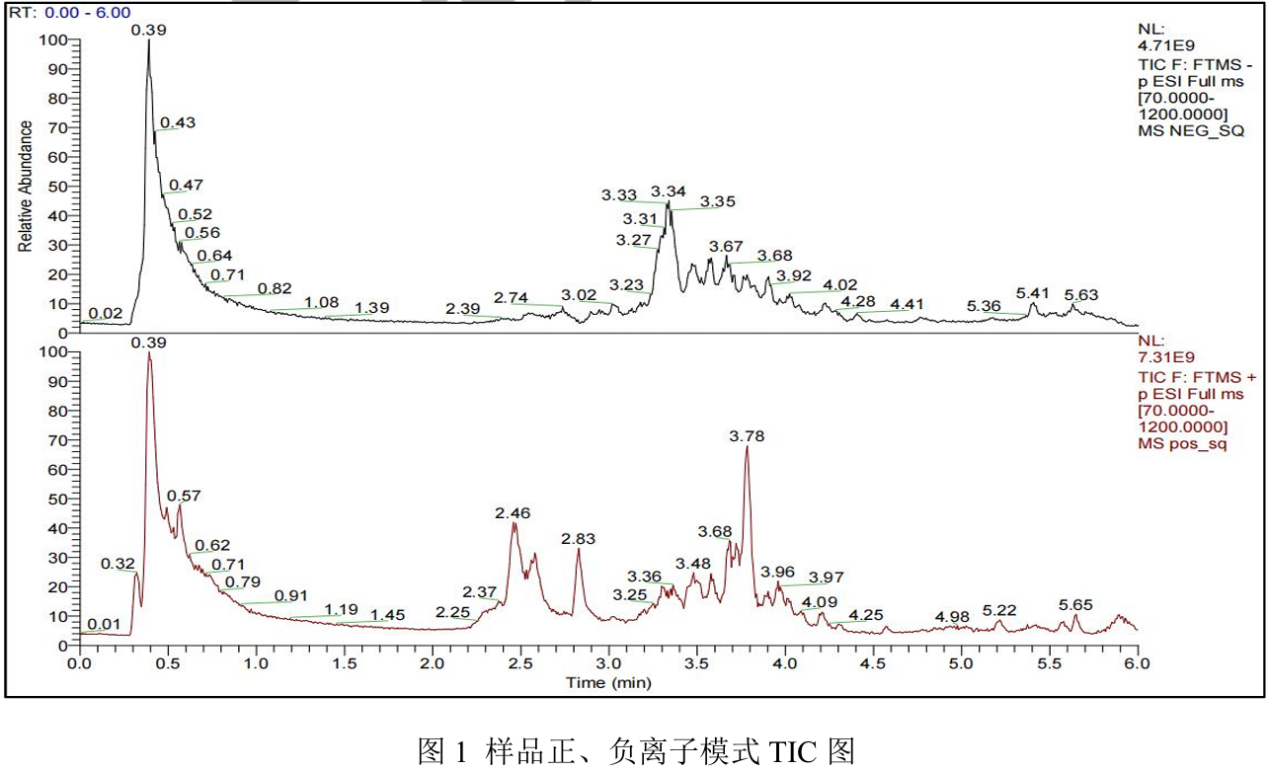


**Figure S1.** LC-MS/MS chromatogram of ARE.


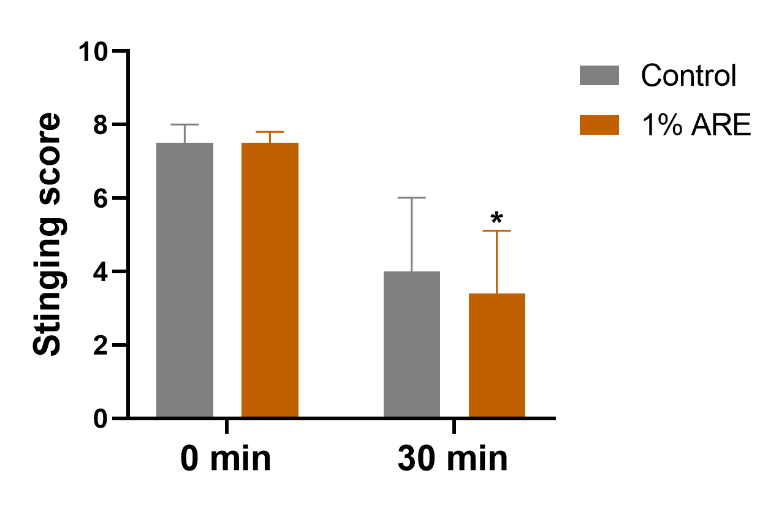


**Figure S2.** Capsaicin-induced stinging scores assessed before and after application of distilled water (control) or a 1.0% ARE formulation. Fifteen Asian adults (18-55 years) participated in the study. The test was conducted on the inner forearm under controlled environmental conditions (21 ± 1 °C, 50 ± 10% relative humidity). A 200 μL aliquot of 0.1% capsaicin in ethanol was applied under occlusion for 10 minutes, followed by removal of the patch. Subjects exhibiting a redness induction rate ≥ 40% were included in the analysis. Each test formulation (distilled water or 1.0% ARE) was applied once (25 μL) to the test site after capsaicin challenge. Stinging intensity was rated on a 0-10 scale at baseline, immediately after capsaicin exposure (*t*_0_), and 30 min after formulation application (*t*_30_).

**Table S1.** Chemical composition of Ashwagandha root extract (ARE) identified by LC-MS/MS

| **No.** | **RT (min)** | **Identified compound** | **Relative content (%)** | **Molecular formula** | CAS No. | **Category** |
| --- | --- | --- | --- | --- | --- | --- |
| 1 | 0.330 | Pyrogallol | 0.111 | C_6_H_6_O_3_ | 87-66-1 | Phenols |
| 2 | 0.397 | Melibiose | 10.096 | C_12_H_22_O_11_ | 13299-20-2 | Saccharides |
| 3 | 0.437 | Isoleucine | 0.867 | C_6_H_13_NO_2_ | 73-32-5 | Amino acids |
| 4 | 0.468 | 2-Hydroxyisobutyric acid | 0.125 | C_4_H_8_O_3_ | 594-61-6 | Organic acids |
| 5 | 0.523 | Threonine | 0.237 | C_4_H_9_NO_3_ | 72-19-5 | Amino acids |
| 6 | 0.565 | Phenylalanine | 1.211 | C_9_H_11_NO_2_ | 63-91-2 | Amino acids |
| 7 | 0.565 | Arecaidine | 0.270 | C_7_H_11_NO_2_ | 499-04-7 | Alkaloids |
| 8 | 0.617 | Glutaric acid | 0.271 | C_5_H_8_O_4_ | 110-94-1 | Organic acids |
| 9 | 0.637 | 3,5-Dimethoxy-4-(β-D-glucopyranosyloxy)benzoic acid | 0.082 | C_15_H_20_O_10_ | 33228-65-8 | Phenylglycosides |
| 10 | 0.753 | 2-Hydroxy-3-methylbutyric acid | 0.466 | C_5_H_10_O_3_ | 600-37-3 | Organic acids |
| 11 | 0.760 | Tryptophan | 0.141 | C_11_H_12_N_2_O_2_ | 73-22-3 | Amino acids |
| 12 | 0.823 | Maltol | 0.271 | C_6_H_6_O_3_ | 118-71-8 | Pyrone derivatives‌ |
| 13 | 0.900 | 2,4-Dihydroxybenzoic acid | 0.352 | C_7_H_6_O_4_ | 89-86-1 | Organic acids |
| 14 | 1.212 | 2-Ketocaproic acid | 0.171 | C_6_H_10_O_3_ | 2492-75-3 | Organic acids |
| 15 | 2.348 | Chlorogenic acid | 0.158 | C_16_H_18_O_9_ | 327-97-9 | Phenols |
| 16 | 2.443 | N-Feruloylputrescine | 0.348 | C_14_H_20_N_2_O_3_ | 501-13-3 | Phenylamides |
| 17 | 2.463 | Caffeine | 0.265 | C_8_H_10_N_4_O_2_ | 58-08-2 | Alkaloids |
| 18 | 2.748 | Piperonylic acid | 0.057 | C_8_H_6_O_4_ | 94-53-1 | Organic acids |
| 19 | 2.752 | Desaminotyrosine | 0.246 | C_9_H_10_O_3_ | 501-97-3 | Organic acids |
| 20 | 3.038 | Suberic acid | 0.880 | C_8_H_14_O_4_ | 505-48-6 | Organic acids |
| 21 | 3.255 | Tropolone | 0.275 | C_7_H_6_O_2_ | 533-75-5 | Tropolones |
| 22 | 3.267 | Ononin | 0.301 | C_22_H_22_O_9_ | 486-62-4 | Flavonoids |
| 23 | 3.277 | Farrerol 4'-O-beta-D-glucopyranoside | 0.087 | C_23_H_26_O_10_ | 1421704-70-2 | Flavonoids |
| 24 | 3.317 | 2,3-Dihydrowithanolide E | 0.142 | C_28_H_40_O_7_ | 38253-76-8 | Withanolides |
| 25 | 3.337 | N-p-trans-Coumaroyltyramine | 0.110 | C_17_H_17_NO_3_ | 36417-86-4 | Phenylamides |
| 26 | 3.345 | Azelaic acid | 7.041 | C_9_H_16_O_4_ | 123-99-9 | Organic acids |
| 27 | 3.380 | Butyl lactate | 0.173 | C_7_H_14_O_3_ | 138-22-7 | Esters |
| 28 | 3.480 | (E)-4-Hydroxydodec-2-enedioic acid | 0.162 | C_12_H_20_O_5_ | — | Organic acids |
| 29 | 3.692 | Withaferin A | 20.517 | C_28_H_38_O_6_ | 5119-48-2 | Withanolides |
| 30 | 3.693 | 5α,6β,17α,27β-Tetrahydroxywithanolide | 0.051 | C_28_H_40_O_7_ | — | Withanolides |
| 31 | 3.803 | Isoformononetin | 0.776 | C_16_H_12_O_4_ | 486-63-5 | Flavonoids |
| 32 | 3.820 | 2,3-Dihydrowithaferin A | 0.802 | C_28_H_40_O_6_ | 5589-41-3 | Withanolides |
| 33 | 3.960 | (2R,6R)-2-Methyl-4-oxo-6-(trihydroxycholestane-derived)heptanoic acid | 0.678 | C_30_H_46_O_7_ | 103773-62-2 | Terpenoids |
| 34 | 3.973 | (E)-9,12,13-Trihydroxyoctadec-10-enoic acid | 2.220 | C_18_H_34_O_5_ | 29907-56-0 | Organic acids |
| 35 | 3.995 | Isoastragaloside IV | 0.063 | C_41_H_68_O_14_ | 136033-55-1 | Terpenoids |
| 36 | 3.998 | Astragaloside III | 0.086 | C_41_H_68_O_14_ | 84687-42-3 | Terpenoids |
| 37 | 4.082 | 2-Hexyl-3-methylene-butanedioic acid | 0.150 | C_11_H_18_O_4_ | 94513-51-6 | Organic acids |
| 38 | 4.250 | Phytosphingosine | 0.081 | C_18_H_39_NO_3_ | 554-62-1 | Sphingoid bases |
| 39 | 4.293 | Soyasaponin I | 0.063 | C_48_H_78_O_18_ | 51330-27-9 | Terpenoids |
| 40 | 4.412 | Pelargonic acid | 0.705 | C_9_H_18_O_2_ | 112-05-0 | Organic acids |
| 41 | 5.032 | Linoleamide | 0.654 | C_18_H_33_NO | 3072-13-7 | Amides |
| 42 | 5.183 | Kaurenoic acid | 0.062 | C_20_H_30_O_2_ | 6730-83-2 | Terpenoids |
| 43 | 5.408 | Palmitic acid | 1.622 | C_16_H_32_O_2_ | 57-10-3 | Organic acids |
| 44 | 5.408 | 6-[(2E)-3,7-dimethylocta-2,6-dienyl]-7-hydroxy-chromen-2-one | 0.189 | C_19_H_22_O_3_ | 148-83-4 | Phenylpropanoids |
| 45 | 5.642 | Stearic acid | 0.830 | C_18_H_36_O_2_ | 57-11-4 | Organic acids |

**Table S2.** Key excipients of the base O/W cream vehicle

| **Category** | **INCI Name** | **Function** |
| --- | --- | --- |
| Solvent | Aqua | Continuous phase |
| Humectants | Glycerin; Butylene glycol; Dipropylene glycol; Betaine; Caprylyl glycol | Skin hydration |
| Chelating agent | Disodium EDTA | Metal ion chelation |
| Rheology modifiers | Carbomer; Acrylates/C10‑30 alkyl acrylate crosspolymer; Polyvinyl alcohol | Thickening and stabilization |
| Emollients | Pentaerythrityl tetraethylhexanoate; Limnanthes alba (meadowfoam) seed oil; Cetyl ethylhexanoate; Cetearyl alcohol; Triethylhexanoin; Dimethicone; Dimethicone/vinyl dimethicone crosspolymer | Skin conditioning and barrier support |
| Emulsifiers | Glyceryl stearate; PEG‑100 stearate; Cetearyl glucoside | O/W emulsion stabilization |
| pH regulator | Arginine | pH adjustment |
| Preservative system | Hydroxyacetophenone; 1,2-Hexanediol | Microbial protection |

**Table S3.** Subjective sensitivity questionnaire scoring criteria.

| **Item** | **0** | **1** | **2** | **3** |
| --- | --- | --- | --- | --- |
| 1. Discomfort (burning, redness, itching, stinging, tightness) during seasonal or temperature changes | Never | Occasionally | Frequently | Every time |
| 2. Redness, itching, stinging, or tightness triggered by physical (e.g., exercise, collision) or emotional (e.g., stress) factors | Never | Occasionally | Frequently | Every time |
| 3. Discomfort (burning, redness, itching, stinging, tightness) triggered by cosmetics or jewelry | Never | Occasionally | Frequently | Every time |
| 4. Self-perceived skin sensitivity | Not sensitive | Mildly sensitive | Moderately sensitive | Highly sensitive |

**Table S4.** Lactic-acid stinging test scoring criteria.

| **Score** | **Description** |
| --- | --- |
| 0 | No stinging sensation |
| 1 | Mild stinging |
| 2 | Moderate stinging |
| 3 | Severe stinging, intolerable |

**Table S5.** Outcome measures and assessment methods.

| **Category** | **Outcome measure** | **Assessment method/Instrument** | **Time points** | **Measurement site** |
| --- | --- | --- | --- | --- |
| Safety and tolerability | Sensitive skin confirmation | 5% lactic-acid stinging test vs. distilled water | Baseline (D_0_) | Nasolabial fold |
|  | Adverse reaction monitoring | Clinical observation (stinging, irritation, erythema) | Throughout study | Whole face |
|  | Tolerance and acceptance | Self-assessment questionnaire | D_7_, D_14_, D_21_, D_28_ | Whole face |
| Instrumental efficacy | Skin hydration | Corneometer® CM825 | D_0_, D_28_ | Cheek |
|  | Skin elasticity (Q1) & firmness (F4) | Cutometer® dual MPA580 | D_0_, D_7_, D_28_ | Cheek |
|  | Wrinkle analysis - crow’s feet and under-eye | PRIMOS-CR (wrinkle number, area, length, depth, roughness Rz) | D_0_, D_7_, D_28_ | Periorbital area |
|  | Wrinkle analysis – nasolabial folds | Antera 3D (length, width, depth, roughness Ra) | D_0_, D_7_, D_28_ | Nasolabial fold |
|  | Facial contour and volume | VECTRA H2 (jawline length, mandibular angle, apple cheek volume) | D_0_, D_7_, D_28_ | Whole face |
|  | Skin surface photography | VISIA 7 multi-light imaging | D_0_, D_7_, D_28_ | Whole face |
